# Prognostic Significance of the Modified Glasgow Prognostic Score in Patients With Stage IV Melanoma Receiving Immune Checkpoint Inhibitors: A Single‐Center Retrospective Study

**DOI:** 10.1111/1346-8138.70131

**Published:** 2026-01-07

**Authors:** Ken Horisaki, Shusuke Yoshikawa, Wataru Omata, Arata Tsutsumida, Yoshio Kiyohara

**Affiliations:** ^1^ Department of Dermatology Shizuoka Cancer Center Shizuoka Japan; ^2^ Department of Dermatology Nagoya University Graduate School of Medicine Nagoya Japan

**Keywords:** immune checkpoint inhibitor, melanoma, modified Glasgow Prognostic Score, prognostic biomarkers, systemic inflammation

## Abstract

Immune checkpoint inhibitors (ICIs) have transformed the treatment landscape for malignant melanoma (MM). However, given variable response rates and potential toxicities, identifying robust biomarkers is crucial. We investigated whether the modified Glasgow Prognostic Score (mGPS) predicts treatment efficacy and toxicity in Asian patients with advanced MM receiving first‐line ICIs. We retrospectively analyzed 132 patients with stage IV MM treated at Shizuoka Cancer Center between 2012 and 2024. Patients were stratified by mGPS, which integrates C‐reactive protein and albumin levels. Outcomes included objective response rate (ORR), progression‐free survival (PFS), overall survival (OS), and the incidence of severe adverse events. The cohort was distributed as mGPS0 (70.5%), mGPS1 (12.9%), and mGPS2 (16.7%). Treatment efficacy declined notably with higher scores: ORR was 28.0% for mGPS0 and 23.5% for mGPS1, but dropped to 4.5% for mGPS2. Survival analysis revealed a clear prognostic stratification; median PFS was 6.2, 2.5, and 1.5 months, and median OS was 23.5, 14.6, and 1.9 months for mGPS0, mGPS1, and mGPS2, respectively. The mGPS2 group demonstrated significantly worse survival outcomes compared to the mGPS0 group. Conversely, the incidence of grade ≥ 3 adverse events did not differ significantly among the categories. The mGPS is a simple, accessible biomarker reflecting systemic inflammation and nutritional status. It effectively predicts ICI treatment outcomes in patients with advanced MM, particularly identifying those with mGPS2 as having a significantly poor prognosis.

## 1 Introduction

Immune checkpoint inhibitors (ICIs) and molecular targeted therapies have transformed the treatment approach for malignant melanoma (MM). However, clinical trials report response rates of approximately 40% for PD‐1 antibody (PD‐1) monotherapy and 50%–60% for combination therapy with PD‐1 and anti‐CTLA4 antibody [1, 2, 3, 4, 5, 6], indicating that many patients still do not benefit from ICIs. ICIs are also associated with substantial toxicity. A pooled analysis reported that 55.5% of patients with advanced MM treated with nivolumab plus ipilimumab (NIVO+IPI) experienced severe adverse events [7]. In addition, ICI therapy has significantly increased cancer treatment costs due to higher drug prices and prolonged treatment duration compared to conventional systemic therapies [8].

Given the variability in efficacy, toxicity, and economic burden, identifying biomarkers to predict ICI benefits in patients with MM is essential.

Scoring systems reflecting systemic inflammation and nutritional status have recently gained attention as potential biomarkers of ICI response. Well‐known indices such as the neutrophil‐lymphocyte ratio (NLR), systemic immune‐inflammation index (SII), controlling nutritional status (CONUT), and the prognostic nutritional index (PNI) have been studied in this context. The Glasgow Prognostic Score (GPS) and its modified version (mGPS) are also included. GPS and mGPS have been reported to be prognostic factors in various malignant diseases treated with ICI [9]. However, the prognostic utility of mGPS in patients with MM receiving ICIs has only been evaluated in one German cohort [10] and has not been evaluated in Asian patients. This validation in an Asian cohort is critical due to significant ethnic and geographical differences in MM characteristics. Unlike Western populations where cutaneous melanoma associated with high tumor mutational burden is predominant, MM in Asian populations is characterized by a higher prevalence of non‐cutaneous subtypes, namely acral lentiginous and mucosal melanoma [11, 12]. These subtypes are generally associated with a lower tumor mutational burden, leading to lower response rates to ICIs and a distinct biological background [11, 12]. Furthermore, the mGPS components (C‐reactive protein [CRP] and albumin [ALB]) reflect systemic inflammation and nutritional status, which may vary across ethnic groups due to differences in nutritional background and underlying health conditions [13, 14, 15], potentially altering the prognostic impact of the score. Therefore, evaluating mGPS is essential to provide clinically relevant, subtype‐specific prognostic information for Asian patients receiving ICI therapy. To address this gap, we investigated whether mGPS predicts treatment efficacy and toxicity in patients with stage IV MM undergoing first‐line ICI therapy.

## 2 Methods

### 2.1 Study Population and Data Collection

We retrospectively analyzed patients with stage IV MM who received first‐line ICI therapy at Shizuoka Cancer Center between February 2012 and December 2024.

The inclusion criteria were as follows: (i) histologically confirmed MM of any subtype; (ii) stage IV classification according to the American Joint Committee on Cancer (AJCC) 8th edition; (iii) treatment with a first‐line ICI, including anti‐PD‐1 monotherapy (nivolumab and pembrolizumab), or combination therapy with both (NIVO+IPI); and (iv) availability of baseline CRP and ALB values before ICI administration. To avoid confounding factors affecting systemic inflammation, patients with active infections requiring antibiotic treatment within 14 days prior to ICI administration or those with chronic inflammatory diseases were excluded. Collected clinical data included age, sex, Eastern Cooperative Oncology Group performance status (ECOG‐PS) score, MM subtype, AJCC stage IV classification details, BRAF mutation status, treatment‐related adverse events (TRAEs), and laboratory values such as CRP, ALB, and lactate dehydrogenase (LDH) at the start of ICI therapy. Specifically, all baseline CRP and ALB values used for mGPS calculation were obtained from blood samples drawn within 7 days prior to the initiation of the first‐line ICI treatment. Clinical staging followed AJCC 8th edition guidelines; for unclassified genital, anal, or urinary sites, staging criteria for cutaneous melanoma were applied.

### 2.2 Definition of the mGPS

The mGPS was calculated using CRP and ALB values to assess inflammation and nutritional status. A score of 0 was assigned when CRP was < 10 mg/L. A score of 1 was assigned when CRP was > 10 mg/L with ALB > 35 g/L. A score of 2 was assigned when the CRP level was > 10 mg/L and ALB was < 35 g/L (Table 1) [16].

**TABLE 1:** Scoring criteria for modified Glasgow Prognostic Score.

CRP and ALB values	mGPS score
CRP ≤ 10 mg/L and any ALB	0
CRP > 10 mg/L and ALB ≥ 35 g/L	1
CRP > 10 mg/L and ALB < 35 g/L	2

Abbreviations: ALB, serum albumin; CRP, C‐reactive protein; mGPS, modified Glasgow Prognostic Score.

### 2.3 Efficacy Assessment

Primary outcomes included objective response rate (ORR), progression‐free survival (PFS), and overall survival (OS) by mGPS score. The secondary outcome was the incidence of grade ≥ 3 TRAEs. Treatment response was evaluated using the Response Evaluation Criteria in Solid Tumors (RECIST) version 1.1. ORR was defined as the proportion of patients achieving either a complete response (CR) or partial response (PR). TRAE severity was graded using the Common Terminology Criteria for Adverse Events (CTCAE) version 5.0.

### 2.4 Statistical Analysis

Baseline characteristics were compared using the Mann–Whitney *U* test for continuous variables and the chi‐square or Fisher's exact test for categorical variables and ORRs across groups. PFS and OS were estimated using the Kaplan–Meier method, and between‐group differences were assessed with the log‐rank test. PFS and OS were defined from the time a first‐line ICI treatment was initiated until radiological or clinical tumor progression (PFS), death from any cause (OS), or final follow‐up (PFS and OS). Independent prognostic factors for PFS and OS were evaluated using Cox proportional hazards regression. Hazard ratios (HRs) with 95% confidence intervals (CIs) were reported. For the multivariate Cox proportional hazards models, missing data were handled using listwise deletion, meaning only patients with complete data for all covariates included in the model (mGPS, age, gender, ECOG‐PS, LDH levels, and ICI regimen) were used for the analysis. Additionally, to compare the prognostic predictive ability of the mGPS with that of the CONUT score, which was previously evaluated in the same patient group [17], we used the Harrell's concordance index (C‐index) for OS. The Harrell C‐index was calculated based on the Cox proportional hazards model and evaluated the model's concordance between the predicted and observed survival times. In the previous evaluation of the CONUT score, the analysis was performed on 123 patients (excluding 9 patients due to missing total cholesterol data). Therefore, to ensure a comparable analysis in the current study, the C‐index for mGPS was also calculated using this cohort of 123 patients. Statistical significance was set at *p* < 0.05. All statistical analyses were performed using EZR (Saitama Medical Center, Jichi Medical University, Saitama, Japan), a graphical interface for R (The R Foundation for Statistical Computing, Vienna, Austria).

### 2.5 Ethics Statement

This retrospective cohort study was approved by the Institutional Review Board of Shizuoka Cancer Center (approval number: J2024‐86). The requirement for informed consent was waived due to the retrospective observational design. All personal data were handled in compliance with the ethical standards of the 1964 Declaration of Helsinki and its later amendments.

## 3 Results

### 3.1 Baseline Demographics

Clinical and demographic characteristics of the 132 patients with stage IV MM are summarized in Table 2. Of the total, 70.5% had mGPS0, 12.9% had mGPS1, and 16.7% had mGPS2. Median follow‐up was 16.0, 14.3, and 1.9 months in the mGPS0, mGPS1, and mGPS2 groups, respectively. The proportion of patients receiving PD‐1 monotherapy versus NIVO+IPI was 77.4% versus 22.6% in the mGPS0 group, 82.4% versus 17.6% in the mGPS1 group, and 90.9% versus 9.1% in the mGPS2 group (*p* = 0.423). No significant differences in age, gender, stage IV subclassification, primary tumor site, or BRAF mutation status were observed across the three groups (*p* = 0.113–0.644). Increasing mGPS score was associated with higher ECOG‐PS (*p* < 0.001) and mortality (*p* = 0.045). LDH levels were particularly low in the mGPS0 group (*p* = 0.001).

**TABLE 2:** Baseline characteristics of patients with stage IV malignant melanoma.

Characteristic	Patient group (%)				*p* value
	Total	mGPS0 *n* = 93	mGPS1 *n* = 17	mGPS2 *n* = 22	
Patients *n* (%)	132 (100)	93 (70.5)	17 (12.9)	22 (16.7)	
Age median (range)	67.5 [27, 87]	67.0 [27.0, 87.0]	65.0 [44.0, 86.0]	68.5 [43.0, 86.0]	0.644
Sex					
Male	74 (56.1)	48 (51.6)	12 (70.6)	14 (63.6)	0.257
Female	58 (43.9)	45 (48.4)	5 (29.4)	8 (36.4)	
ECOG‐PS score					
0–1	120 (90.9)	91 (97.8)	14 (82.4)	15 (68.2)	**< 0.001** *
≧ 2	12 (9.1)	2 (2.2)	3 (17.6)	7 (31.8)	
Primary site					
Cutaneous without acral	45 (34.1)	31 (33.3)	6 (35.3)	8 (36.4)	0.113
Acral	21 (15.9)	13 (14.0)	1 (5.9)	7 (31.8)	
Mucosal	48 (36.4)	37 (39.8)	7 (41.2)	4 (18.2)	
Uveal	9 (6.8)	8 (8.6)	0 (0.0)	1 (4.5)	
Unknown	9 (6.8)	4 (4.3)	3 (17.6)	2 (9.1)	
Details of Stage IV					
M1a	7 (5.3)	6 (6.5)	1 (5.9)	0 (0.0)	0.358
M1b	24 (18.2)	21 (22.6)	2 (11.8)	1 (4.5)	
M1c	88 (66.7)	58 (62.4)	12 (70.6)	18 (81.8)	
M1d	13 (9.8)	8 (8.6)	2 (11.8)	3 (13.6)	
Liver metastases					
Yes	50 (37.9)	31 (33.3)	7 (41.2)	12 (54.5)	0.174
LDH value					
< ULN	66 (50.0)	56 (60.2)	4 (23.5)	6 (27.3)	**0.001** *
≥ ULN	66 (50.0)	37 (39.8)	13 (76.5)	16 (72.7)	
*BRAF*					
Mutant	19 (14.4)	12 (12.9)	4 (23.5)	3 (13.6)	0.527
Wild	80 (60.6)	59 (63.4)	7 (41.2)	14 (63.6)	
Not investigated	33 (25.0)	22 (23.7)	6 (35.3)	5 (22.7)	
ICI for first‐line treatment					
Anti‐PD‐1 antibody monotherapy	98 (74.2)	72 (77.4)	11 (64.7)	15 (68.2)	0.423
Nivolumab + Ipilimumab	34 (25.8)	21 (22.6)	6 (35.3)	7 (31.8)	
Outcome					
Dead	96 (72.7)	62 (66.7)	14 (82.4)	20 (90.9)	**0.045** *
Alive	36 (27.3)	31 (33.3)	3 (17.6)	2 (9.1)	

Abbreviations: ECOG‐PS, Eastern Cooperative Oncology Group Performance Status; ICI, immune checkpoint inhibitor; LDH, lactate dehydrogenase; mGPS, modified Glasgow Prognostic Score; TRAE, treatment‐related adverse event; ULN, upper limit of normal.

* Bold letters indicate statistically significant differences: *p* < 0.05.

### 3.2 Objective Response

ORRs were 28.0% in the mGPS0 group, 23.5% in the mGPS1 group, and 4.5% in the mGPS2 group (Table 3). The mGPS0 group had a significantly better ORR than the mGPS2 group (*p* = 0.012), but the differences between mGPS0 and mGPS1 (*p* = 0.772) and between mGPS1 and mGPS2 (*p* = 0.147) were not significant. In a sub‐analysis of patients treated with PD‐1 inhibitors, ORRs were 29.1% in the mGPS0 group, 18.2% in the mGPS1 group, and 0.0% in the mGPS2 group (Table S1). Consistent with the overall trend, the mGPS0 group showed a significantly better ORR than the mGPS2 group (*p* = 0.017), but the differences between mGPS0 and mGPS1 (*p* = 0.719) and between mGPS1 and mGPS2 (*p* = 0.169) were not significant. Among patients treated with NIVO+IPI, ORRs were 23.8% for mGPS0, 33.3% for mGPS1, and 14.3% in the mGPS2 group (Table S2). There was no significant difference in ORR between any two groups: mGPS0 and mGPS1 (*p* = 0.633), mGPS0 and mGPS2 (*p* = 1.000), or mGPS1 and mGPS2 (*p* = 0.559).

**TABLE 3:** Objective response rates by mGPS groups.

	Patient group (%)				*p* value
	Total *n* = 132	mGPS0 *n* = 93	mGPS1 *n* = 17	mGPS2 *n* = 22	
Best overall response					
Complete response	6 (4.5)	6 (6.5)	0 (0.0)	0 (0.0)	**< 0.001** *
Partial response	25 (18.9)	20 (21.5)	4 (23.5)	1 (4.5)	
Stable disease	34 (25.8)	29 (31.2)	4 (23.5)	1 (4.5)	
Progressive disease	67 (50.8)	38 (40.9)	9 (52.9)	20 (91.0)	
ORR	31 (23.4)	26 (28.0)	4 (23.5)	1 (4.5)	**0.037** *

Abbreviations: mGPS, modified Glasgow Prognostic Score; ORR, objective response rate.

* Bold letters indicate statistically significant differences: *p* < 0.05.

### 3.3 PFS and OS

**FIGURE 1:**
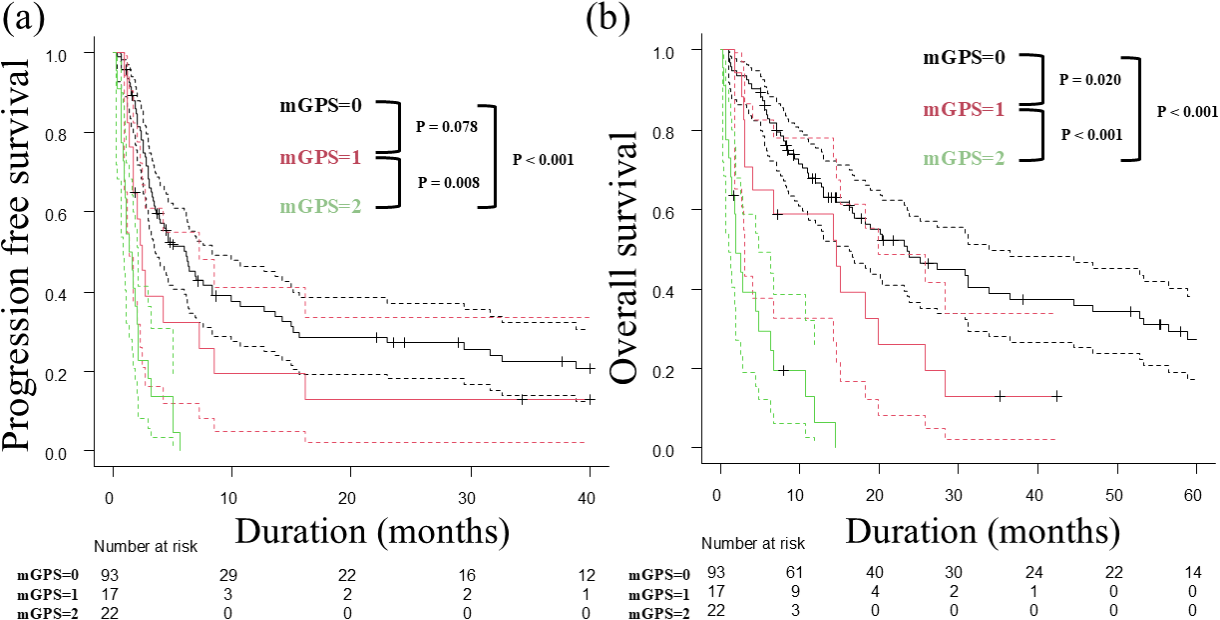
Kaplan–Meier curves for progression‐free survival (PFS) and overall survival (OS) according to modified Glasgow Prognostic Score (mGPS). (a) PFS by the mGPS group. Median PFS is 6.2, 2.5, and 1.5 months for mGPS0, mGPS1, and mGPS2, respectively. PFS is significantly shorter in mGPS2 compared to mGPS0 (*p* < 0.001) and mGPS1 (*p* = 0.008), and also significantly shorter in mGPS1 than mGPS0 (*p* = 0.078). (b) OS by the mGPS group. Median OS is 23.5, 14.6, and 1.9 months for mGPS0, mGPS1, and mGPS2, respectively. OS is significantly worse in mGPS2 compared to both mGPS0 (*p* < 0.001) and mGPS1 (*p* < 0.001), and also significantly worse in mGPS1 than mGPS0 (*p* = 0.010).

Median PFS and OS significantly decreased with higher mGPS scores across the entire cohort (PFS: 6.2–1.5 months; OS: 23.5–1.9 months) (Figure 1). Similar trends were observed in the PD‐1 subgroup (Figure S1). In the NIVO+IPI subgroup, the mGPS2 group had significantly worse PFS and OS than the mGPS0 group (Figure S2). In addition, in a sub‐analysis of patients with an ECOG‐PS of 1 or higher, the mGPS2 group had significantly worse PFS and OS than the mGPS0 group (Figure S3).

### 3.4 Multivariate Analysis for PFS and OS Rate and C‐Index

A multivariate analysis was performed, adjusting for age, gender, ECOG‐PS, LDH levels and ICI regimens (Table 4). For PFS, mGPS2 (reference mGPS0, HR: 4.360, 95% CI: 2.437–7.800, *p* < 0.001) and ECOG‐PS ≥ 2 (reference ECOG‐PS ≤ 1, HR: 2.144, 95% CI: 1.040–4.420, *p* = 0.039) were the independent predictors. For OS, mGPS2 (reference mGPS0, HR: 6.164, 95% CI: 3.176–11.96, *p* < 0.001) was the only independent predictor. In a direct comparison of the prognostic predictive ability of mGPS and CONUT scores for OS using Harrell's C‐index, mGPS (C‐index: 0.653) had slightly better overall survival discriminatory ability than CONUT (C‐index: 0.633).

**TABLE 4:** Multivariate Cox proportional hazards model for progression‐free and overall survival in patients with stage IV malignant melanoma.

	Progression‐free survival			Overall survival		
	Hazard ratio	95% CI	*p* value	Hazard ratio	95% CI	*p* value
mGPS						
mGPS0	Reference			Reference		
mGPS1	1.341	0.691–2.601	0.386	1.609	0.827–3.13	0.162
mGPS2	4.360	2.437–7.800	**< 0.001** *	6.164	3.176–11.96	**< 0.001** *
Sex						
Female	Reference			Reference		
Male	1.273	0.851–1.905	0.240	1.139	0.726–1.787	0.570
Age	1.013	0.995–1.031	0.147	1.014	0.993–1.035	0.186
ECOG‐PS						
score 0–1	Reference			Reference		
score ≥ 2	2.144	1.040–4.420	**0.039** *	1.803	0.846–3.842	0.127
LDH value < ULN	Reference			Reference		
≥ ULN	0.867	0.563–1.336	0.519	1.023	0.642–1.630	0.922
ICI for first‐line treatment						
Nivolumab + Ipilimumab	Reference			Reference		
Anti‐PD‐1 antibody monotherapy	0.686	0.422–1.114	0.128	0.653	0.388–1.098	0.108

Abbreviation: CI, confidence interval.

* Bold letters indicate statistically significant differences: *p* < 0.05.

### 3.5 Toxicity

Grade ≥ 3 TRAEs resulting in treatment discontinuation occurred in 18.2% of all patients (Table S3). The incidence of grade ≥ 3 TRAEs was 21.5% in the mGPS0 group, 17.6% in the mGPS1 group, and 4.5% in the mGPS2 group. There was no significant difference in the incidence of grade ≥ 3 TRAEs between mGPS0 and mGPS1 (*p* = 1.000), mGPS1 and mGPS2 (*p* = 0.300), or mGPS0 and mGPS2 (*p* = 0.072).

## 4 Discussion

This is the first study to examine the prognostic value of the mGPS in Asian patients with MM receiving ICIs as first‐line therapy. The main findings were as follows: (i) ORR, PFS, and OS worsened with increasing mGPS; (ii) similar trends were observed in the PD‐1 subgroup; (iii) mGPS was an independent predictor of PFS and OS; and (iv) mGPS was not significantly associated with grade ≥ 3 TRAEs.

The mGPS, based on CRP and ALB, reflects systemic inflammation and nutritional status. CRP is a well‐established marker of systemic inflammation and has been implicated in promoting immune suppression, tumor progression, and metastasis [18]. CRP has also been shown to directly suppress T‐ and dendritic cells, thereby impairing the efficacy of ICIs by dampening innate and adaptive immune responses in patients with cancer [19, 20]. Han et al. conducted a meta‐analysis of 6124 patients with cancer receiving ICI and found that patients with pretreatment CRP > 10 mg/L had significantly worse PFS and OS [21]. Serum ALB is routinely used to assess nutritional status, disease severity, and prognosis, as its synthesis is suppressed by both malnutrition and inflammation [22]. Accordingly, numerous studies have explored the prognostic role of ALB in malignancies [23, 24, 25], and recent evidence suggests that hypoalbuminemia is a poor prognostic factor in patients with cancer receiving ICI therapy [26, 27]. Turner et al. reported that pembrolizumab clearance is accelerated in cachectic states, including hypoalbuminemia, and that this is associated with reduced OS in patients with advanced MM and non‐small cell lung cancer (NSCLC) [28]. This suggests that the systemic inflammation and hypercatabolic state reflected by the mGPS may alter ICI pharmacokinetics, leading to accelerated clearance of monoclonal antibodies and reduced therapeutic exposure. Given their prognostic factors, mGPS, which incorporates CRP and ALB, may serve as a more comprehensive predictor of outcomes in patients receiving ICIs. Mechanistically, the prognostic utility of mGPS reflects the biological activity of proinflammatory cytokines, particularly interleukin‐6 (IL‐6). As highlighted by Fisher et al. [29], IL‐6 acts as a key driver of the hepatic acute‐phase response, which directly upregulates CRP production while suppressing ALB synthesis. Beyond these systemic effects, IL‐6 plays a critical role in orchestrating an immunosuppressive tumor microenvironment. It promotes the recruitment and expansion of myeloid‐derived suppressor cells and inhibits the cytotoxic function of effector T cells, thereby creating a significant barrier to the efficacy of ICIs. Although individual components such as CRP, ALB, and LDH have prognostic value, the mGPS integrates the inflammatory and nutritional status into a validated scoring system. This integration captures the synergistic impact of cancer cachexia more robustly than single markers and facilitates comparison with previous international studies. In addition, considering that CRP and ALB are produced in the liver, hepatic metastases could theoretically affect the mGPS classification. However, in our cohort, there was no significant difference in the incidence of liver metastases among the mGPS groups (Table 2). This suggests that the prognostic value of mGPS observed in this study is likely reflective of systemic inflammation and nutritional status rather than being solely a surrogate for hepatic tumor burden.

Zhang et al. conducted a meta‐analysis of 963 patients with various cancers treated with ICIs and found that high mGPS was significantly associated with poorer PFS and OS [9]. The meta‐analysis included patients with NSCLC, renal cell carcinoma, urothelial carcinoma, gastric cancer, head and neck cancer, and salivary gland cancer, with each study reporting a significant prognostic association for mGPS. Regarding MM, Pflug et al. investigated the prognostic value of mGPS in 284 German patients with cutaneous MM [10]. A sub‐analysis of 117 patients who received first‐line ICI monotherapy showed that mGPS2 (median OS: 1.6 months) had a significantly worse OS than mGPS0 (median OS: not reached) and mGPS1 (median OS: not reached) (*p* < 0.001), but there was little difference in OS between mGPS0 and mGPS1 (2‐year OS rate: 65.8% vs. 64.8%). Furthermore, multivariate analysis showed that mGPS was an independent prognostic factor for PFS (*p* < 0.001) and OS (*p* < 0.001). Overall, the results of this study were generally similar to those of the previous German study, but the prognosis of the mGPS1 group receiving ICI monotherapy was significantly worse than in the German study (2‐year OS rate: 18.2% in this study vs. 64.8% in the German study). The present study included Asian patients, who are less likely to respond to ICIs, and more than half of the patients had subtypes other than primary cutaneous MM. Furthermore, the mGPS1 group had a higher incidence of elevated LDH levels than the German study (76.5% in this study vs. 63% in the German study). These findings may explain the discrepancies in the conclusions regarding the prognosis of mGPS0 and mGPS1 patients between the two studies. The prognosis of patients with MM treated with ICIs may be significantly affected by differences in race, subtype, and treatment regimen, and prognostic markers should be considered separately for each individual condition [30, 31]. Although this is the second study to evaluate the prognostic value of mGPS in MM, the findings, in light of prior studies, support its potential as a universal prognostic biomarker across cancer types in the context of ICI therapy. Furthermore, there are often clinical situations where clinicians hesitate to administer ICI therapy to patients with poor ECOG‐PS. In this study, even in the subgroup analysis of patients with ECOG‐PS ≥ 1, poor mGPS showed a tendency for worse PFS and OS. While the decision to administer ICI should be based on a combination of subjective and objective factors, mGPS may be useful as a reference finding.

In MM, other biomarkers, such as NLR, SII, and CONUT score have been investigated for prognostic value in patients treated with ICI. However, the prognostic relevance of NLR and SII remains controversial, as some studies report significant associations [32, 33, 34, 35, 36, 37, 38, 39], while others do not [40, 41, 42]. Inconsistencies in cutoff values across studies and the variability of NLR/SII due to non‐cancer‐related conditions have limited their clinical utility. Since categorical indices pose fewer challenges with cutoff selection than continuous variables like NLR and SII, we previously evaluated the CONUT score as a prognostic marker in patients with advanced MM receiving first‐line ICI therapy [17]. The CONUT score integrates ALB, total cholesterol, and lymphocyte count to reflect nutritional and inflammatory status. We previously found that patients with CONUT scores ≥ 3 had significantly poorer ORR (*p* = 0.020), PFS (*p* = 0.017), and OS (*p* < 0.001) compared to those with scores ≤ 2 group. Furthermore, in this study, we compared the prognostic predictive ability of mGPS and CONUT based on Harrell's C‐index, and found that although mGPS was slightly superior, there was almost no difference in their predictive ability. Although CONUT and mGPS differ in their components beyond ALB, their similar prognostic value likely reflects their shared ability to capture systemic inflammation and nutritional status. To our knowledge, as these are the only studies to assess both CONUT and mGPS in Asian patients with MM, further research is needed to compare the prognostic accuracy of these scores, along with NLR and SII, in patients with MM treated with ICI.

We compared the incidence of grade ≥ 3 TRAEs across mGPS groups. Although the differences were not statistically significant, the incidence of grade ≥ 3 TRAEs decreased with increasing mGPS (Table S3). A similar pattern was observed in our previous CONUT study where the incidence of grade ≥ 3 TRAEs was 20.9% in the CONUT ≤ 2 group versus 16.1% in the CONUT ≥ 3 group (*p* = 0.643) [17]. Given the results of both efficacy and side effects, it can be inferred that the activation of autoimmunity by ICI is less likely to occur in high inflammation and low nutritional status. Supporting this observation, a meta‐analysis of 11 491 patients with cancer reported a significantly higher incidence of immune‐related adverse events in those with low NLR, reflecting lower systemic inflammation (OR, 0.55, *p* < 0.001) [43]. However, it cannot be ruled out that the incidence of immune‐related adverse events may be underestimated in patients with high inflammation and poor nutritional status due to their poor prognosis and shorter follow‐up period.

This study identified mGPS as a potential prognostic scoring system for patients with MM undergoing ICI therapy. However, several limitations should be acknowledged. First, the retrospective cohort design may have introduced selection bias. Second, the single‐center design and limited sample size, particularly in the NIVO+IPI subgroup, may have limited the power of statistical analysis. Third, while this was a validation cohort for previous studies evaluating mGPS in MM, it is the first derivation cohort in Asian patients. Therefore, external validation in an independent cohort is necessary before clinical implementation. Fourth, although short follow‐up generally introduces bias into the assessment of OS, we acknowledge that the short median follow‐up time observed in the mGPS2 group (1.9 months) reflects the aggressive nature of the disease and the low efficacy of ICI in this cohort. The high rate of events (20 deaths out of 22 patients) in this group underscores this finding.

In conclusion, mGPS may serve as a prognostic indicator in patients with stage IV MM receiving first‐line ICI therapy. Our preliminary findings from a retrospective, single‐institutional cohort suggest that mGPS is a useful tool. However, before its routine clinical application, our results require rigorous prospective validation in larger, multi‐center, and ethnically diverse cohorts. Furthermore, future research should focus on comparing its utility with other established scoring systems.

## Ethics Statement

Reviewed and approved by the Ethics Committee of Shizuoka Cancer Center: approval no. #J2024‐86.

## Consent

The authors have nothing to report.

## Conflicts of Interest

The authors declare no conflicts of interest.

## Supporting information


**Figure S1:** Kaplan–Meier curves for progression‐free survival (PFS) and overall survival (OS) according to modified Glasgow Prognostic Score (mGPS) in patients receiving PD‐1 monotherapy. (a) PFS by mGPS group. Median PFS was 6.9, 2.7 and 1.7 months for mGPS0, mGPS1, and mGPS2, respectively. PFS was significantly shorter in mGPS2 compared to mGPS0 (*p* < 0.001) and mGPS1 (*p* = 0.013), but there was no significant difference between mGPS0 and mGPS1 (*p* = 0.110). (b) OS by mGPS group. Median OS was 23.5, 14.3, and 28.3 months for mGPS0, mGPS1, and mGPS2, respectively. OS was significantly worse in mGPS2 compared to both mGPS0 (*p* < 0.001) and mGPS1 (*p* = 0.010), and also significantly worse in mGPS1 than mGPS0 (*p* = 0.011).

**Figure S2:** Kaplan–Meier curves for progression‐free survival (PFS) and overall survival (OS) according to modified Glasgow Prognostic Score (mGPS) in patients receiving a combination therapy with nivolumab plus ipilimumab. (a) PFS by mGPS group. Median PFS was 4.6, 1.7 and 1.1 months for mGPS0, mGPS1, and mGPS2, respectively. PFS was significantly shorter in mGPS2 compared to mGPS0 (*p* = 0.006), but there was no significant difference between mGPS0 and mGPS1 (*p* = 0.430), or between mGPS1 and mGPS2 (*p* = 0.290). (b) OS by mGPS group. Median OS was 14.7, 15.2, and 1.9 months for mGPS0, mGPS1, and mGPS2, respectively. OS was significantly worse in mGPS2 compared to both mGPS0 (*p* < 0.001) and mGPS1 (*p* = 0.019), but there was no significant difference between mGPS0 and mGPS1 (*p* = 0.685).

**Figure S3:** Kaplan–Meier curves for progression‐free survival (PFS) and overall survival (OS) according to modified Glasgow Prognostic Score (mGPS) in patients with ECOG‐PS ≥ 1. (a) PFS by mGPS group. Median PFS was 6.3, 2.1 and 1.1 months for mGPS0, mGPS1, and mGPS2, respectively. PFS was significantly shorter in mGPS2 compared to mGPS0 (*p* < 0.001), but there was no significant difference between mGPS0 and mGPS1 (*p* = 0.977), or between mGPS1 and mGPS2 (*p* = 0.079). (b) OS by mGPS group. Median OS was 18.8, 5.4, and 1.7 months for mGPS0, mGPS1, and mGPS2, respectively. OS was significantly worse in mGPS2 compared to mGPS0 (*p* < 0.001), but there was no significant difference between mGPS0 and mGPS1 (*p* = 0.650), or between mGPS1 and mGPS2 (*p* = 0.129).

**Table S1:** Objective response rates by mGPS groups receiving PD‐1 monotherapy.
**Table S2:** Objective response rates by mGPS groups receiving NIVO+IPI.
**Table S3:** Grade ≥ 3 treatment‐related adverse events leading to first‐line therapy discontinuation, stratified by mGPS group (*N* = 132).

## Data Availability

The data that support the findings of this study are available from the corresponding author upon reasonable request.

## References

[1] N. Yamazaki , T. Takenouchi , M. Fujimoto , et al., “Phase 1b Study of Pembrolizumab (MK‐3475; Anti‐PD‐1 Monoclonal Antibody) in Japanese Patients With Advanced Melanoma (KEYNOTE‐041),” Cancer Chemotherapy and Pharmacology 79, no. 4 (2017): 651–660, 10.1007/s00280-016-3237-x. 28283736 PMC5364262

[2] C. Robert , J. Schachter , G. V. Long , et al., “Pembrolizumab Versus Ipilimumab in Advanced Melanoma,” New England Journal of Medicine 372, no. 26 (2015): 2521–2532, 10.1056/NEJMoa1503093. 25891173

[3] J. Larkin , V. Chiarion‐Sileni , R. Gonzalez , et al., “Combined Nivolumab and Ipilimumab or Monotherapy in Untreated Melanoma,” New England Journal of Medicine 373, no. 1 (2015): 23–34, 10.1056/NEJMx180040. 26027431 PMC5698905

[4] A. Ribas , O. Hamid , A. Daud , et al., “Association of Pembrolizumab With Tumor Response and Survival Among Patients With Advanced Melanoma,” JAMA 315, no. 15 (2016): 1600–1609, 10.1001/jama.2016.6779. 27092830

[5] M. C. T. van Zeijl , J. van Breeschoten , L. C. de Wreede , et al., “Real‐World Outcomes of Ipilimumab Plus Nivolumab Combination Therapy in a Nation‐Wide Cohort of Advanced Melanoma Patients in The Netherlands,” Journal of Immunotherapy 46, no. 5 (2023): 197–204, 10.1097/CJI.0000000000000468. 37103470

[6] F. S. Hodi , J. Chesney , A. C. Pavlick , et al., “Combined Nivolumab and Ipilimumab Versus Ipilimumab Alone in Patients With Advanced Melanoma: 2‐Year Overall Survival Outcomes in a Multicentre, Randomised, Controlled, Phase 2 Trial,” Lancet Oncology 17, no. 11 (2016): 1558–1568, 10.1016/S1470-2045(16)30366-7. 27622997 PMC5630525

[7] M. Sznol , P. F. Ferrucci , D. Hogg , et al., “Pooled Analysis Safety Profile of Nivolumab and Ipilimumab Combination Therapy in Patients With Advanced Melanoma,” Journal of Clinical Oncology 35 (2017): 3815–3822, 10.1200/JCO.2016.72.1167. 28915085

[8] A. Vergnenegre and C. Chouaid , “Economic Analyses of Immune‐Checkpoint Inhibitors to Treat Lung Cancer,” Expert Review of Pharmacoeconomics & Outcomes Research 21, no. 3 (2021): 365–371, 10.1080/14737167.2021.1863790. 33306411

[9] Y. Zhang , S. Chen , H. Chen , and W. Li , “A Comprehensive Analysis of Glasgow Prognostic Score (GPS)/the Modified Glasgow Prognostic Score (mGPS) on Immune Checkpoint Inhibitor Efficacy Among Patients With Advanced Cancer,” Cancer Medicine 12, no. 1 (2023): 38–48, 10.1002/cam4.4940. 35702873 PMC9844653

[10] N. Pflug , M. Vitus , J. Knuever , et al., “Treatment‐Specific Evaluation of the Modified Glasgow‐Prognostic‐Score in Patients With Advanced Cutaneous Melanoma,” Journal of the European Academy of Dermatology and Venereology 35, no. 12 (2021): e879–e883, 10.1111/jdv.17533. 34310762

[11] T. Hida , M. Idogawa , J. Kato , et al., “Genetic Characteristics of Cutaneous, Acral, and Mucosal Melanoma in Japan,” Cancer Medicine 13 (2024): e70360, 10.1002/cam4.70360. 39564955 PMC11577301

[12] X. Bai , A. N. Shoushtari , A. Betof Warner , et al., “Benefit and Toxicity of Programmed Death‐1 Blockade Vary by Ethnicity in Patients With Advanced Melanoma: An International Multicentre Observational Study,” British Journal of Dermatology 187, no. 3 (2022): 401–410, 10.1111/bjd.21241. 35293617

[13] A. Kelley‐Hedgepeth , D. M. Lloyd‐Jones , A. Colvin , et al., “Ethnic Differences in C‐Reactive Protein Concentrations,” Clinical Chemistry 54, no. 6 (2008): 1027–1037, 10.1373/clinchem.2007.098996. 18403563

[14] M. A. Albert , R. J. Glynn , J. Buring , and P. M. Ridker , “C‐Reactive Protein Levels Among Women of Various Ethnic Groups Living in the United States (From the Women's Health Study),” American Journal of Cardiology 93, no. 15 (2004): 1238–1242, 10.1016/j.amjcard.2004.01.067. 15135696

[15] E. Lim , J. Miyamura , and J. J. Chen , “Racial/Ethnic‐Specific Reference Intervals for Common Laboratory Tests: A Comparison Among Asians, Blacks, Hispanics, and White,” Hawai'i Journal of Medicine & Public Health 74, no. 9 (2015): 302–310. 26468426 PMC4578165

[16] M. J. Proctor , D. S. Morrison , D. Talwar , et al., “An Inflammation‐Based Prognostic Score (mGPS) Predicts Cancer Survival Independent of Tumour Site: A Glasgow Inflammation Outcome Study,” British Journal of Cancer 104, no. 4 (2011): 726–734, 10.1038/sj.bjc.6606087. 21266974 PMC3049591

[17] K. Horisaki , S. Yoshikawa , S. Mori , W. Omata , A. Tsutsumida , and Y. Kiyohara , “Prognostic Value of the CONUT Score With Immune Checkpoint Inhibitors as First‐Line Therapy for Metastatic Malignant Melanoma,” Journal of Dermatology 52, no. 4 (2025): 615–623, 10.1111/1346-8138.17613. 39916640 PMC11975212

[18] T. L. Whiteside , “The Tumor Microenvironment and Its Role in Promoting Tumor Growth,” Oncogene 27, no. 45 (2008): 5904–5912, 10.1038/onc.2008.271. 18836471 PMC3689267

[19] M. A. Bilen , D. J. Martini , Y. Liu , et al., “The Prognostic and Predictive Impact of Inflammatory Biomarkers in Patients Who Have Advanced‐Stage Cancer Treated With Immunotherapy,” Cancer 125, no. 1 (2019): 127–134, 10.1002/cncr.31778. 30329148

[20] N. R. Sproston and J. J. Ashworth , “Role of C‐Reactive Protein at Sites of Inflammation and Infection,” Frontiers in Immunology 9 (2018): 754, 10.3389/fimmu.2018.00754. 29706967 PMC5908901

[21] C. L. Han , G. X. Meng , Z. N. Ding , et al., “The Predictive Potential of the Baseline C‐Reactive Protein Levels for the Efficiency of Immune Checkpoint Inhibitors in Cancer Patients: A Systematic Review and Meta‐Analysis,” Frontiers in Immunology 13 (2022): 827788, 10.3389/fimmu.2022.827788. 35211122 PMC8861087

[22] J. Y. Yeun and G. A. Kaysen , “Factors Influencing Serum Albumin in Dialysis Patients,” American Journal of Kidney Diseases 32, no. 4 (1998): S118–S125, 10.1016/s0272-6386(98)70174-x. 9892378

[23] Z. H. Yao , G. Y. Tian , S. X. Yang , et al., “Serum Albumin as a Significant Prognostic Factor in Patients With Malignant Pleural Mesothelioma,” Tumour Biology 35, no. 7 (2014): 6839–6845, 10.1007/s13277-014-1938-5. 25051913

[24] D. Gupta and C. G. Lis , “Pretreatment Serum Albumin as a Predictor of Cancer Survival: A Systematic Review of the Epidemiological Literature,” Nutrition Journal 9 (2010): 69, 10.1186/1475-2891-9-69. 21176210 PMC3019132

[25] K. Miura , K. Hamanaka , T. Koizumi , et al., “Clinical Significance of Preoperative Serum Albumin Level for Prognosis in Surgically Resected Patients With Non‐Small Cell Lung Cancer: Comparative Study of Normal Lung, Emphysema, and Pulmonary Fibrosis,” Lung Cancer 111 (2017): 88–95, 10.1016/j.lungcan.2017.07.003. 28838406

[26] Y. Guo , L. Wei , S. H. Patel , et al., “Serum Albumin: Early Prognostic Marker of Benefit for Immune Checkpoint Inhibitor Monotherapy but Not Chemoimmunotherapy,” Clinical Lung Cancer 23, no. 4 (2022): 345–355, 10.1016/j.cllc.2021.12.010. 35131184 PMC9149057

[27] Y. Saito , K. Kobayashi , O. Fukuoka , et al., “Ultra‐High Combined Positive Score and High Serum Albumin Are Favorable Prognostic Biomarkers for Immune Checkpoint Inhibitors in Head and Neck Squamous Cell Carcinoma,” Head & Neck 46, no. 2 (2024): 367–377, 10.1002/hed.27576. 38063247

[28] D. C. Turner , A. G. Kondic , K. M. Anderson , et al., “Pembrolizumab Exposure‐Response Assessments Challenged by Association of Cancer Cachexia and Catabolic Clearance,” Clinical Cancer Research 24, no. 23 (2018): 5841–5849, 10.1158/1078-0432.CCR-18-0415. 29891725

[29] D. T. Fisher , M. M. Appenheimer , and S. S. Evans , “The Two Faces of IL‐6 in the Tumor Microenvironment,” Seminars in Immunology 26, no. 1 (2014): 38–47, 10.1016/j.smim.2014.01.008. 24602448 PMC3970580

[30] E. Rossi , G. Schinzari , B. A. Maiorano , et al., “Efficacy of Immune Checkpoint Inhibitors in Different Types of Melanoma,” Human Vaccines & Immunotherapeutics 17, no. 1 (2021): 4–13, 10.1080/21645515.2020.1771986. 32663057 PMC7872095

[31] K. Namikawa , T. Ito , S. Yoshikawa , et al., “Systemic Therapy for Asian Patients With Advanced BRAF V600‐Mutant Melanoma in a Real‐World Setting: A Multi‐Center Retrospective Study in Japan (B‐CHECK‐RWD Study),” Cancer Medicine 12, no. 17 (2023): 17967, 10.1002/cam4.6438. 37584204 PMC10524053

[32] M. Capone , D. Giannarelli , D. Mallardo , et al., “Baseline Neutrophil‐To‐Lymphocyte Ratio (NLR) and Derived NLR Could Predict Overall Survival in Patients With Advanced Melanoma Treated With Nivolumab,” Journal for Immunotherapy of Cancer 6, no. 1 (2018): 74, 10.1186/s40425-018-0383-1. 30012216 PMC6048712

[33] D. B. Sacdalan , J. A. Lucero , and D. L. Sacdalan , “Prognostic Utility of Baseline Neutrophil‐To‐Lymphocyte Ratio in Patients Receiving Immune Checkpoint Inhibitors: A Review and Meta‐Analysis,” Oncotargets and Therapy 11 (2018): 955–965, 10.2147/OTT.S153290. 29503570 PMC5827677

[34] M. Z. Afzal , T. Sarwar , and K. Shirai , “Prognostic Significance of Hematological Indices in Malignant Melanoma Treated With Immune Checkpoint Inhibitors,” Journal of Immunotherapy 42, no. 7 (2019): 251–264, 10.1097/CJI.0000000000000272. 31145229

[35] A. Kartolo , R. Holstead , S. Khalid , et al., “Serum Neutrophil‐To‐Lymphocyte Ratio and Platelet‐To‐Lymphocyte Ratio in Prognosticating Immunotherapy Efficacy,” Immunotherapy 12, no. 11 (2020): 785–798, 10.2217/imt-2020-0105. 32657234

[36] Y. Matsumura , Y. Kawarada , M. Matsuo , et al., “Retrospective Analysis of Neutrophil‐To‐Lymphocyte Ratio in Patients With Melanoma Who Received Ipilimumab Monotherapy or Ipilimumab in Combination With Nivolumab in Japan,” Biological & Pharmaceutical Bulletin 46, no. 3 (2023): 427–431, 10.1248/bpb.b22-00750. 36858571

[37] S. Anpalakhan , P. Huddar , R. Behrouzi , et al., “Immunotherapy‐Related Adverse Events in Real‐World Patients With Advanced Non‐Small Cell Lung Cancer on Chemoimmunotherapy: A SPINNAKER Study Sub‐Analysis,” Frontiers in Oncology 13 (2023): 1163768, 10.3389/fonc.2023.1163768. 37324003 PMC10265987

[38] J. H. Lee , S. Hyung , J. Lee , and S. H. Choi , “Visceral Adiposity and Systemic Inflammation in the Obesity Paradox in Patients With Unresectable or Metastatic Melanoma Undergoing Immune Checkpoint Inhibitor Therapy: A Retrospective Cohort Study,” Journal for Immunotherapy of Cancer 10, no. 8 (2022): e005226, 10.1136/jitc-2022-005226. 36002189 PMC9413167

[39] T. Mesti , C. Grašič Kuhar , and J. Ocvirk , “Biomarkers for Outcome in Metastatic Melanoma in First‐Line Treatment With Immune Checkpoint Inhibitors,” Biomedicine 11, no. 3 (2023): 749, 10.3390/biomedicines11030749. PMC10044937 36979727

[40] K. Kudura , L. Nussbaumer , R. Foerster , and L. Basler , “Inflammatory Blood Parameters as Biomarkers for Response to Immune Checkpoint Inhibition in Metastatic Melanoma Patients,” Biomedicine 10, no. 9 (2022): 2135, 10.3390/biomedicines10092135. PMC9496082 36140238

[41] L. Susok , S. Said , D. Reinert , et al., “The Pan‐Immune‐Inflammation Value and Systemic Immune‐Inflammation Index in Advanced Melanoma Patients Under Immunotherapy,” Journal of Cancer Research and Clinical Oncology 148, no. 11 (2022): 3103–3108, 10.1007/s00432-021-03878-y. 35006344 PMC9508007

[42] Y. Nakamura , S. Kitano , A. Takahashi , et al., “Nivolumab for Advanced Melanoma: Pretreatment Prognostic Factors and Early Outcome Markers During Therapy,” Oncotarget 7, no. 7 (2016): 77404–77415, 10.18632/oncotarget.12677. 27764805 PMC5363594

[43] W. Zhang , Y. Tan , Y. Li , and J. Liu , “Neutrophil to Lymphocyte Ratio as a Predictor for Immune‐Related Adverse Events in Cancer Patients Treated With Immune Checkpoint Inhibitors: A Systematic Review and Meta‐Analysis,” Frontiers in Immunology 14 (2023): 1234142, 10.3389/fimmu.2023.1234142. 37622124 PMC10445236

