# Supplementary figures and images for: Prognostic Significance of the Modified Glasgow Prognostic Score in Patients With Stage IV Melanoma Receiving Immune Checkpoint Inhibitors: A Single‐Center Retrospective Study
Source: J Dermatol. 2026 Jan 7;53(4):587–95. doi: 10.1111/1346-8138.70131 (PMC13075527; doi:10.1111/1346-8138.70131)

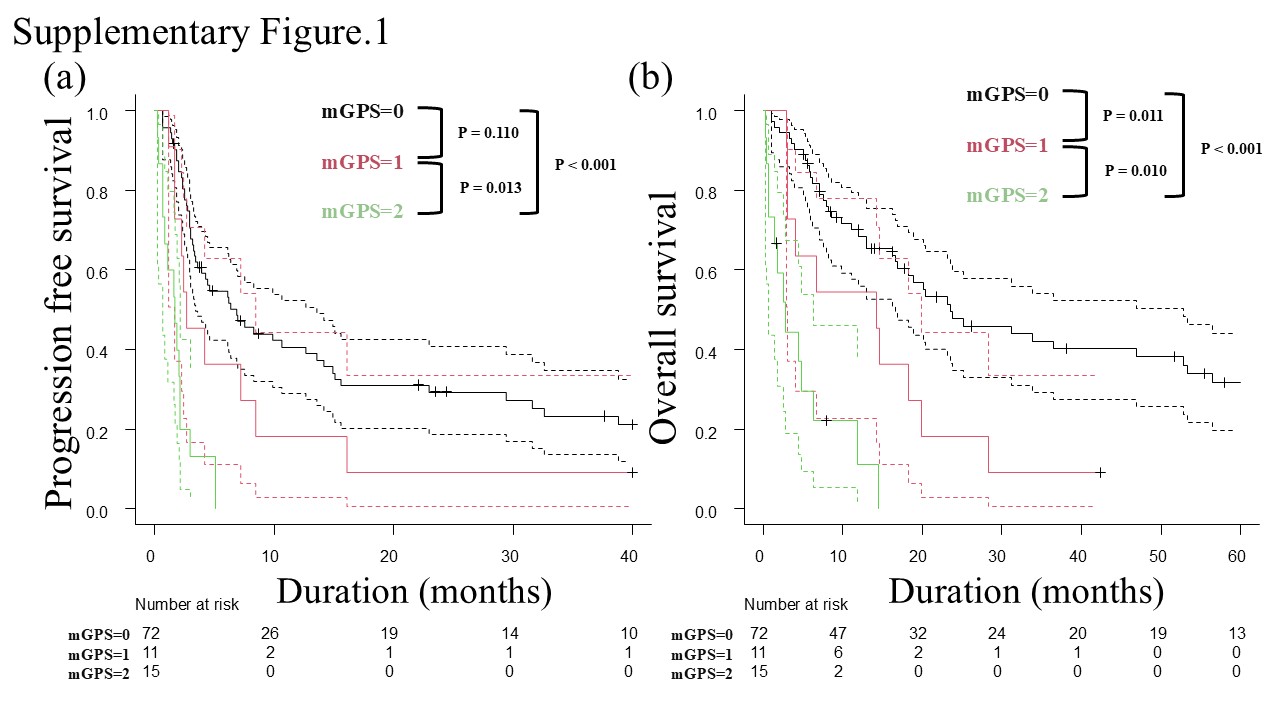

Supplement: Supplementary file 1 — Figure S1: Kaplan–Meier curves for progression‐free survival (PFS) and overall survival (OS) according to modified Glasgow Prognostic Score (mGPS) in patients receiving PD‐1 monotherapy. (a) PFS by mGPS group. Median PFS was 6.9, 2.7 and 1.7 months for mGPS0, mGPS1, and mGPS2, respectively. PFS was significantly shorter in mGPS2 compared to mGPS0 (p < 0.001) and mGPS1 (p = 0.013), but there was no significant difference between mGPS0 and mGPS1 (p = 0.110). (b) OS by mGPS group. Median OS was 23.5, 14.3, and 28.3 months for mGPS0, mGPS1, and mGPS2, respectively. OS was significantly worse in mGPS2 compared to both mGPS0 (p < 0.001) and mGPS1 (p = 0.010), and also significantly worse in mGPS1 than mGPS0 (p = 0.011). [file JDE-53-587-s003.jpg]

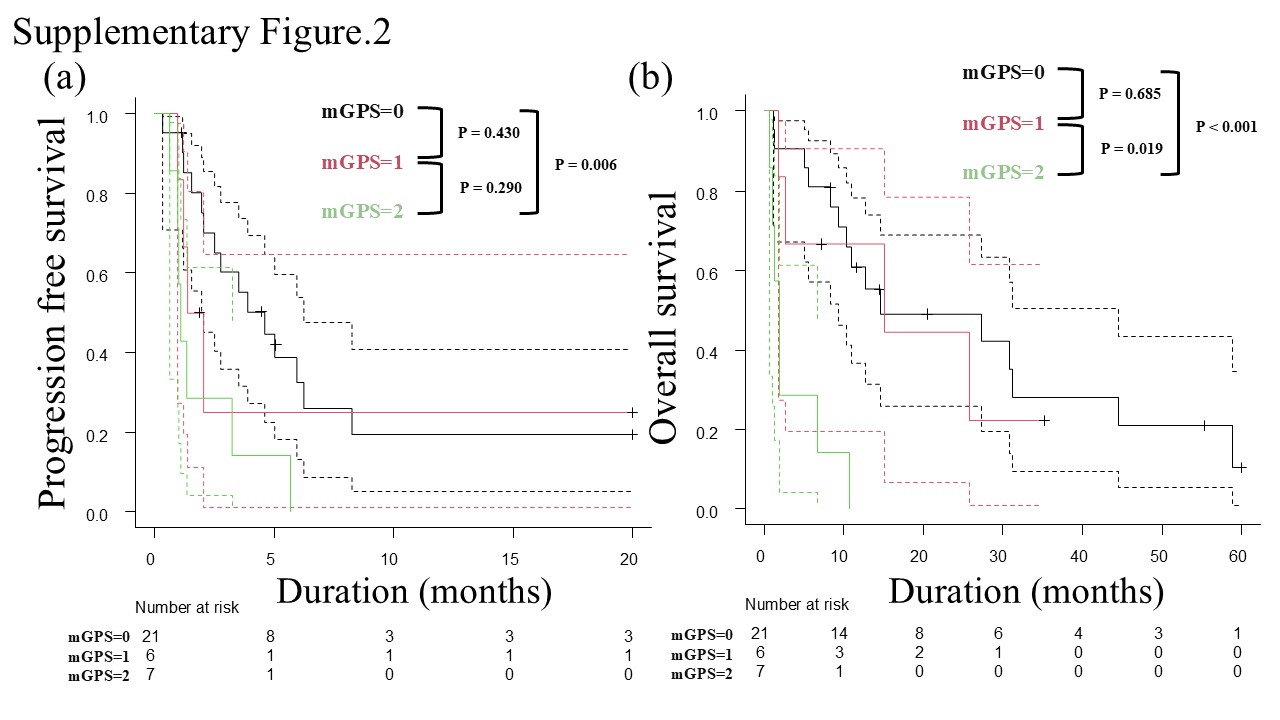

Supplement: Supplementary file 2 — Figure S2: Kaplan–Meier curves for progression‐free survival (PFS) and overall survival (OS) according to modified Glasgow Prognostic Score (mGPS) in patients receiving a combination therapy with nivolumab plus ipilimumab. (a) PFS by mGPS group. Median PFS was 4.6, 1.7 and 1.1 months for mGPS0, mGPS1, and mGPS2, respectively. PFS was significantly shorter in mGPS2 compared to mGPS0 (p = 0.006), but there was no significant difference between mGPS0 and mGPS1 (p = 0.430), or between mGPS1 and mGPS2 (p = 0.290). (b) OS by mGPS group. Median OS was 14.7, 15.2, and 1.9 months for mGPS0, mGPS1, and mGPS2, respectively. OS was significantly worse in mGPS2 compared to both mGPS0 (p < 0.001) and mGPS1 (p = 0.019), but there was no significant difference between mGPS0 and mGPS1 (p = 0.685). [file JDE-53-587-s004.jpg]

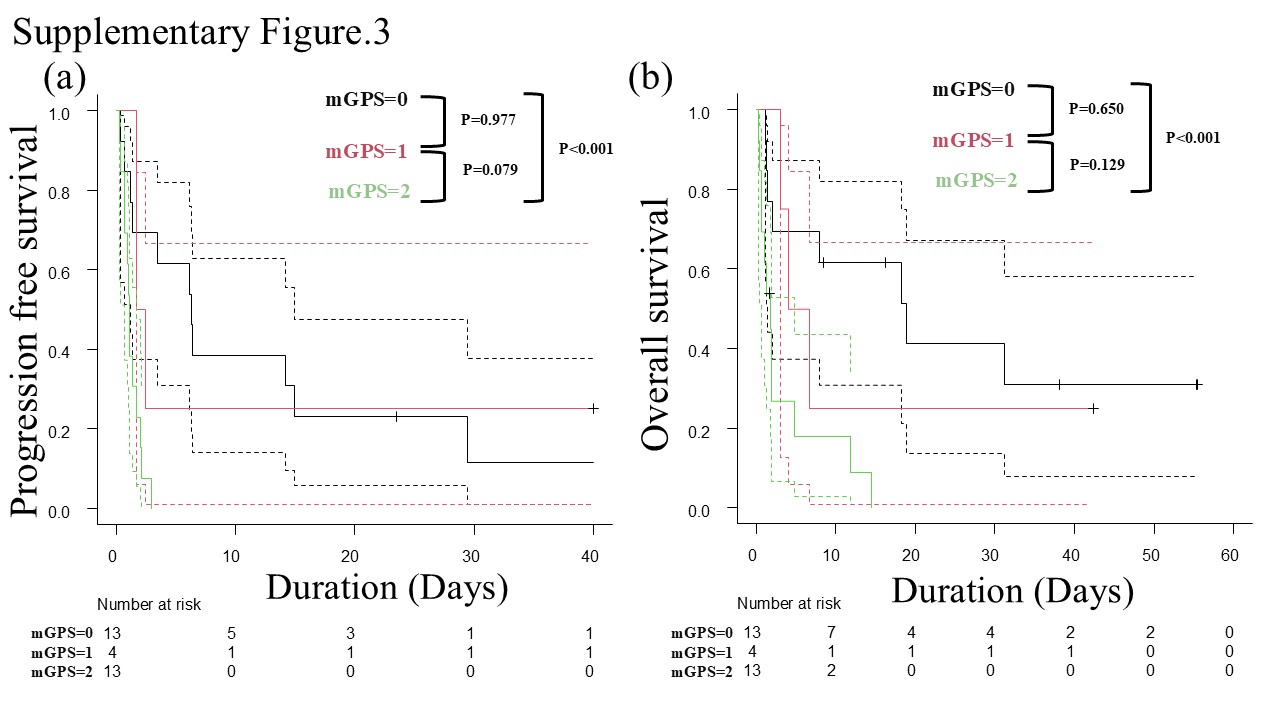

Supplement: Supplementary file 3 — Figure S3: Kaplan–Meier curves for progression‐free survival (PFS) and overall survival (OS) according to modified Glasgow Prognostic Score (mGPS) in patients with ECOG‐PS ≥ 1. (a) PFS by mGPS group. Median PFS was 6.3, 2.1 and 1.1 months for mGPS0, mGPS1, and mGPS2, respectively. PFS was significantly shorter in mGPS2 compared to mGPS0 (p < 0.001), but there was no significant difference between mGPS0 and mGPS1 (p = 0.977), or between mGPS1 and mGPS2 (p = 0.079). (b) OS by mGPS group. Median OS was 18.8, 5.4, and 1.7 months for mGPS0, mGPS1, and mGPS2, respectively. OS was significantly worse in mGPS2 compared to mGPS0 (p < 0.001), but there was no significant difference between mGPS0 and mGPS1 (p = 0.650), or between mGPS1 and mGPS2 (p = 0.129). [file JDE-53-587-s002.jpg]
