# Supplementary material for: Prognostic Significance of the Modified Glasgow Prognostic Score in Patients With Stage IV Melanoma Receiving Immune Checkpoint Inhibitors: A Single‐Center Retrospective Study
Source: J Dermatol. 2026 Jan 7;53(4):587–95. doi: 10.1111/1346-8138.70131 (PMC13075527; doi:10.1111/1346-8138.70131)
Supplement: Supplementary file 4 — Table S1: Objective response rates by mGPS groups receiving PD‐1 monotherapy. Table S2: Objective response rates by mGPS groups receiving NIVO+IPI. Table S3: Grade ≥ 3 treatment‐related adverse events leading to first‐line therapy discontinuation, stratified by mGPS group (N = 132). [file JDE-53-587-s001.docx]

**Supplementary Figure 1. Kaplan-Meier curves for progression-free survival (PFS) and overall survival (OS) according to modified Glasgow Prognostic Score (mGPS) in patients receiving PD-1 monotherapy.** (a) PFS by mGPS group. Median PFS was 6.9, 2.7 and 1.7 months for mGPS0, mGPS1, and mGPS2, respectively. PFS was significantly shorter in mGPS2 compared to mGPS0 (P < 0.001) and mGPS1 (P = 0.013), but there was no significant difference between mGPS0 and mGPS1 (P = 0.110). (b) OS by mGPS group. Median OS was 23.5, 14.3, and 28.3 months for mGPS0, mGPS1, and mGPS2, respectively. OS was significantly worse in mGPS2 compared to both mGPS0 (P < 0.001) and mGPS1 (P = 0.010), and also significantly worse in mGPS1 than mGPS0 (P = 0.011).

**Supplementary Figure 2. Kaplan-Meier curves for progression-free survival (PFS) and overall survival (OS) according to modified Glasgow Prognostic Score (mGPS) in patients receiving a combination therapy with nivolumab plus ipilimumab.** (a) PFS by mGPS group. Median PFS was 4.6, 1.7 and 1.1 months for mGPS0, mGPS1, and mGPS2, respectively. PFS was significantly shorter in mGPS2 compared to mGPS0 (P = 0.006), but there was no significant difference between mGPS0 and mGPS1 (P = 0.430), or between mGPS1 and mGPS2 (P = 0.290). (b) OS by mGPS group. Median OS was 14.7, 15.2, and 1.9 months for mGPS0, mGPS1, and mGPS2, respectively. OS was significantly worse in mGPS2 compared to both mGPS0 (P < 0.001) and mGPS1 (P = 0.019), but there was no significant difference between mGPS0 and mGPS1 (P = 0.685).

**Supplementary Figure 3. Kaplan-Meier curves for progression-free survival (PFS) and overall survival (OS) according to modified Glasgow Prognostic Score (mGPS) in patients with ECOG-PS≥1.** (a) PFS by mGPS group. Median PFS was 6.3, 2.1 and 1.1 months for mGPS0, mGPS1, and mGPS2, respectively. PFS was significantly shorter in mGPS2 compared to mGPS0 (P < 0.001), but there was no significant difference between mGPS0 and mGPS1 (P = 0.977), or between mGPS1 and mGPS2 (P = 0.079). (b) OS by mGPS group. Median OS was 18.8, 5.4, and 1.7 months for mGPS0, mGPS1, and mGPS2, respectively. OS was significantly worse in mGPS2 compared to mGPS0 (P < 0.001), but there was no significant difference between mGPS0 and mGPS1 (P = 0.650), or between mGPS1 and mGPS2 (P = 0.129).

**Supplementary Table 1.** Objective response rates by mGPS groups receiving PD-1 monotherapy

|  | Patient group (%) | | | | ***p-value*** |
| --- | --- | --- | --- | --- | --- |
|  | Total  n=98 | mGPS0  n=72 | mGPS1  n=11 | mGPS2  n=15 |  |
| Best overall response |  |  |  |  | **0.014** |
| Complete response | 5 (5.1) | 5 (6.9) | 0 (0.0) | 0 (0.0) |  |
| Partial response | 18 (18.4) | 16 (22.2) | 2 (18.2) | 0 (0.0) |  |
| Stable disease | 28 (28.6) | 23 (31.9) | 4 (36.4) | 1 (6.7) |  |
| Progressive disease | 47 (48.0) | 28 (38.9) | 5 (45.5) | 14 (93.3) |  |
| ORR | 23 (23.5) | 21 (29.1) | 2 (18.2) | 0 (0.0) |  |

mGPS, modified Glasgow Prognostic Score; ORR, objective response rate.

*Bold letters indicate statistically significant differences: P<0.05

**Supplementary Table 2.** Objective response rates by mGPS groups receiving NIVO+IPI

|  | Patient group (%) | | | | ***p-value*** |
| --- | --- | --- | --- | --- | --- |
|  | Total  n=34 | mGPS0  n=21 | mGPS1  n=6 | mGPS2  n=7 |  |
| Best overall response |  |  |  |  | 0.386 |
| Complete response | 1 (2.9) | 1 (4.8) | 0 (0.0) | 0 (0.0) |  |
| Partial response | 7 (20.6) | 4 (19.0) | 2 (33.3) | 1 (14.3) |  |
| Stable disease | 6 (17.6) | 6 (28.6) | 0 (0.0) | 0 (0.0) |  |
| Progressive disease | 20 (58.8) | 10 (47.6) | 4 (66.7) | 6 (85.7) |  |
| ORR | 8 (23.5) | 5 (23.8) | 2 (33.3) | 1 (14.3) |  |

mGPS, modified Glasgow Prognostic Score; NIVO+IPI, nivolumab plus ipilimumab; ORR, objective response rate.

*Bold letters indicate statistically significant differences: P<0.05

**Supplementary Table 3.** Grade ≥3 treatment-related adverse events leading to first-line therapy discontinuation, stratified by mGPS group (N = 132)

| Event | Patients group (%) | | | | *p-value* |
| --- | --- | --- | --- | --- | --- |
|  | Total  N =132 | mGPS0  N = 93 | mGPS1  N = 17 | mGPS2  N = 22 |  |
| TRAE ≥ grade3 | 24 (18.2) | 20 (21.5) | 3 (17.6) | 1 (4.5) | 0.179 |
| Pneumonia | 9 (6.8) | 7 (7.5) | 2 (11.8) | 0 (0.0) | 0.311 |
| Hepatitis | 7 (5.3) | 6 (6.5) | 0 (0.0) | 1 (4.5) | 0.543 |
| Colitis | 2 (1.5) | 2 (2.2) | 0 (0.0) | 0 (0.0) | 0.653 |
| Pituitary dysfunction | 2 (1.5) | 2 (2.2) | 0 (0.0) | 0 (0.0) | 0.653 |
| Hyperglycemia | 1 (0.8) | 1 (1.1) | 0 (0.0) | 0 (0.0) | 0.810 |
| Myositis | 1 (0.8) | 1 (1.1) | 0 (0.0) | 0 (0.0) | 0.810 |
| Neutropenia | 1 (0.8) | 1 (1.1) | 0 (0.0) | 0 (0.0) | 0.810 |
| Hypothyroidism | 1 (0.8) | 1 (1.1) | 0 (0.0) | 0 (0.0) | 0.810 |
| Fever | 1 (0.8) | 0 (0.0) | 1 (5.9) | 0 (0.0) | **0.033*** |

mGPS, modified Glasgow Prognostic Score; TRAE, treatment-related adverse event. Adverse events were graded according to CTCAE v5.0.
*Bold letters indicate statistical significance (P < 0.05)
